# Timely Management of Simultaneous Bilateral Spontaneous Pneumothorax: A Near-Death Experience

**DOI:** 10.7759/cureus.34684

**Published:** 2023-02-06

**Authors:** Shilpa A Gaidhane, Nitish Batra, Apoorva Nirmal, Iftekhar Ansari

**Affiliations:** 1 Internal Medicine, Jawaharlal Nehru Medical College, Datta Meghe Institute of Higher Education and Research, Wardha, IND

**Keywords:** sub pleural bull, life threatening presentation, case report, tuberculosis, simultaneous bilateral spontaneous pneumothorax

## Abstract

Simultaneous bilateral spontaneous pneumothorax is a situation that rarely occurs. The patient can have various presentations, from dyspnoea and chest pain to significant respiratory failure. Although it causes lung collapse in nearly two thirds of cases, early diagnosis and treatment are of the utmost importance. Here, we present the case of an 18-year-old boy who presented with complaints of sudden onset respiratory distress. He was immediately put on mechanical ventilation. He was diagnosed with simultaneous bilateral spontaneous pneumothorax on chest X-ray. He needed bilateral intra-thoracic drainage, following which a video-assisted thoracoscopy was done on the left side.

## Introduction

Spontaneous unilateral pneumothorax is one of the common causes of sudden onset dyspnoea presenting in the emergency department. However, the overall incidence of simultaneous bilateral spontaneous pneumothorax ranges from 1.4% to 7.6% [1]. The clinical presentation varies from mild dyspnoea to a tension pneumothorax causing respiratory failure and death. Bilateral tension pneumothorax can be defined as a case where no tracheal deviation is detected on the chest X-ray and the signs may be equal bilaterally [2]. Here, we report a near-death case with simultaneous bilateral spontaneous pneumothorax due to tuberculosis and rupture of the emphysematous bullae, presenting with respiratory distress and cardiorespiratory arrest in a rural tertiary care hospital.

## Case presentation

**Figure 1:**
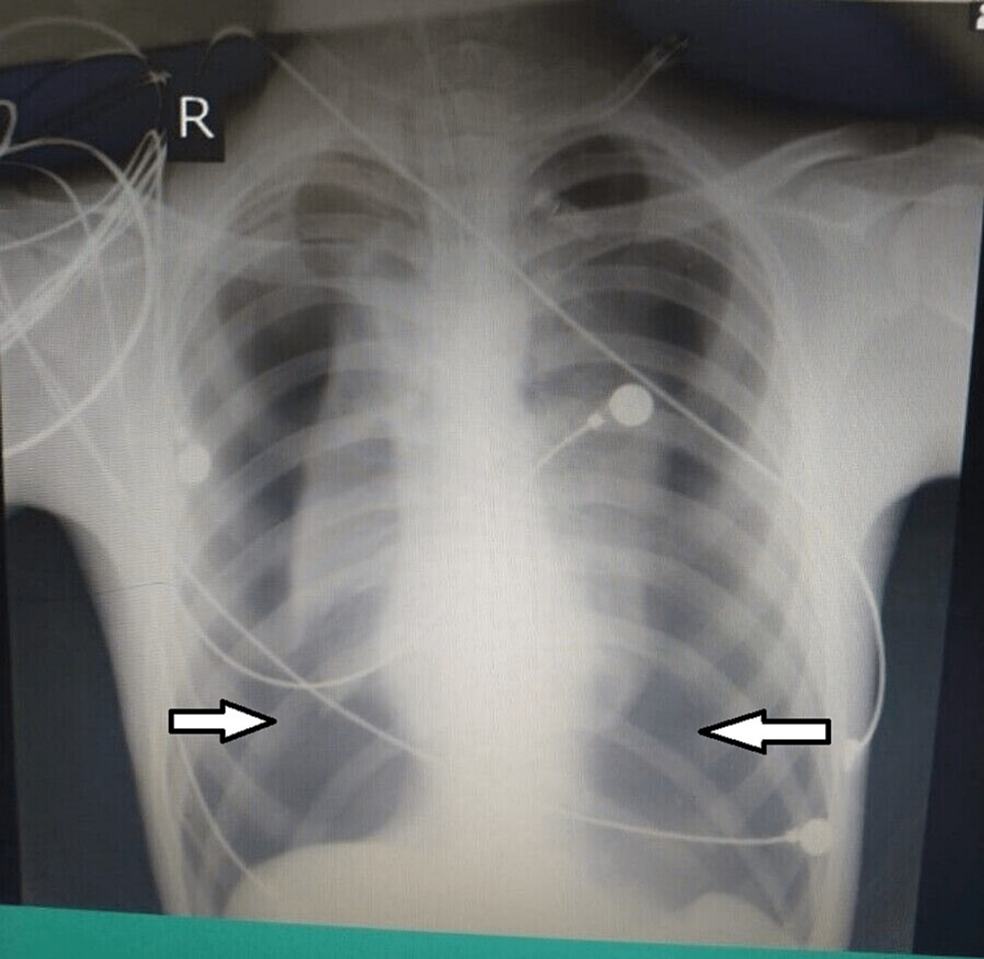
Chest X-Ray showing bilateral pneumothorax

An 18-year-old male was brought to the emergency department with sudden onset shortness of breath for the last thirty minutes. On admission, he had been conscious for five minutes, and his Glasgow Coma Scale (GCS) was E4 V4 M5. Oxygen saturation was 70% on room air, and he was started on 15 litres of nasal oxygen. His blood pressure was 110/70. He was shifted to the medical intensive care unit (MICU). The relatives gave a history of sudden onset breathlessness with no precipitating factor. Relatives took him to a private hospital, where he was initially treated for severe acute asthma due to the absence of breath sounds on both sides. We considered him a case of acute onset respiratory distress, the causes of which are foreign body aspiration, acute pulmonary oedema, pulmonary embolism, pneumothorax, and severe acute asthma. Our patient was young, tall, lean, and thin. On arriving at the MICU, the patient had tachycardia and tachypnoea. His systolic BP had fallen to 80 mmHg with 60% saturation, and bilateral air entry was absent. Arterial blood gas (ABG) showed severe hypoxia even on oxygen therapy. His chest x-ray showed bilateral pneumothorax (Figure 1).

**Figure 2:**
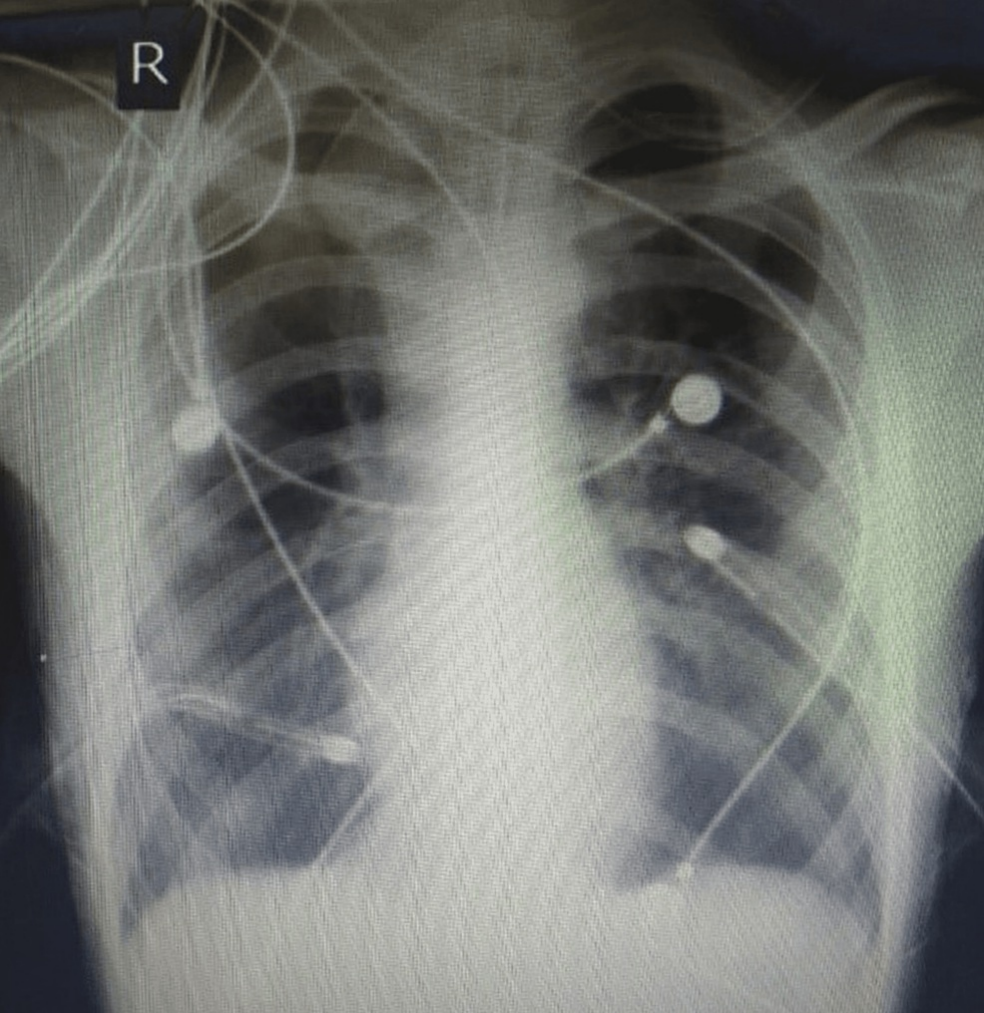
Post ICD insertion, the X-ray showed full expansion on the right side and to some extent, on the left side

Immediately, a 16G Venflon needle (a 5- to 8-cm-long angiocatheter, single-use, sterile) was inserted into the second intercostal space on the mid-clavicular line bilaterally to decrease intrathoracic pressure and expand the lungs. His ABG, performed after needle insertion, showed dramatic improvement, increasing his oxygen saturation (SPO2) from 60% to 90%. The patient was intubated and put on a mechanical ventilator in volume control mode. During intubation, he developed ventricular tachycardia and was therefore given cardioversion. The MICU team revived him after two cycles of cardiopulmonary resuscitation (CPR). The respiratory consultant inserted an intercostal drain (ICD) bilaterally. Immediately after the ICD insertion, the X-ray showed full expansion on the right side and, to some extent, on the left side (Figure 2).

**Figure 3:**
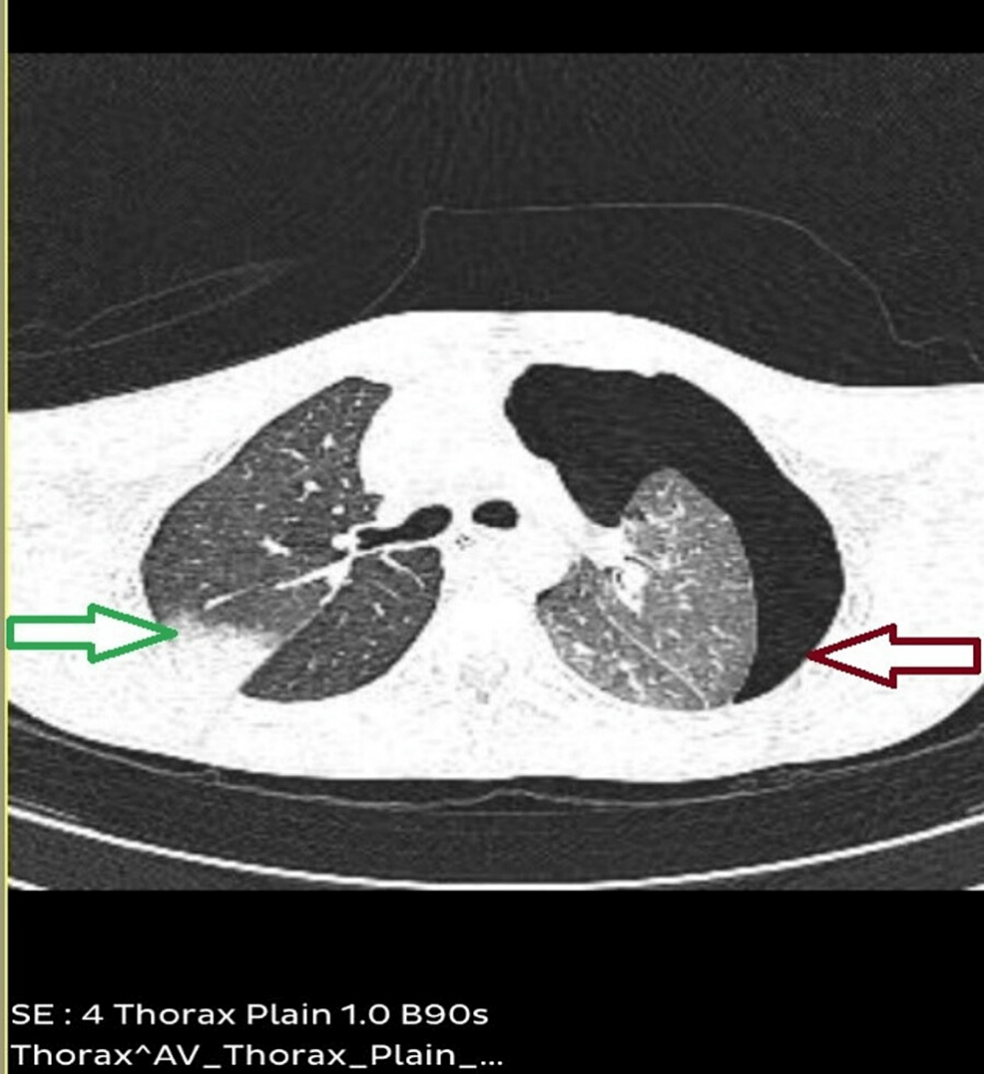
HRCT of the thorax showing consolidation on the left side (green arrow) and pneumothorax on the right side (red arrow)

There was evidence of a broncho-pleural fistula in the left lung, as there was excessive frothing and bubbling of the water column on the left side. Laboratory findings showed a raised leucocyte count, and the rest of the other parameters were regular. The high-resolution computed tomography (HRCT) of the thorax was suggestive of minimal pneumothorax on the right side and pneumothorax with partial collapse of the left lung. It also showed multiple subpleural bullae in both lungs with a wedge-shaped pneumonic patch on the left lower lobe (Figure 3).

**Figure 4:**
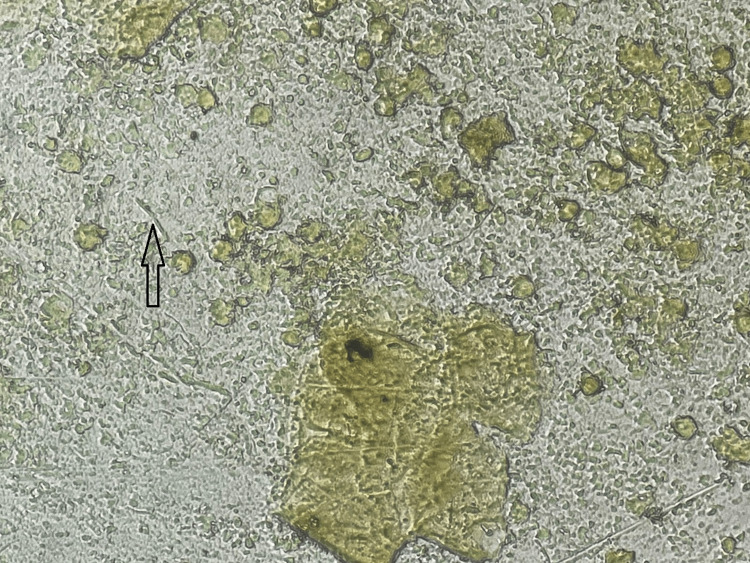
Auramine and rhodamine stain showing fluorescent rod-shaped bacilli

The sputum sample was examined microscopically, and it was positive for Mycobacterium tuberculosis (Figure 4).

**Figure 5:**
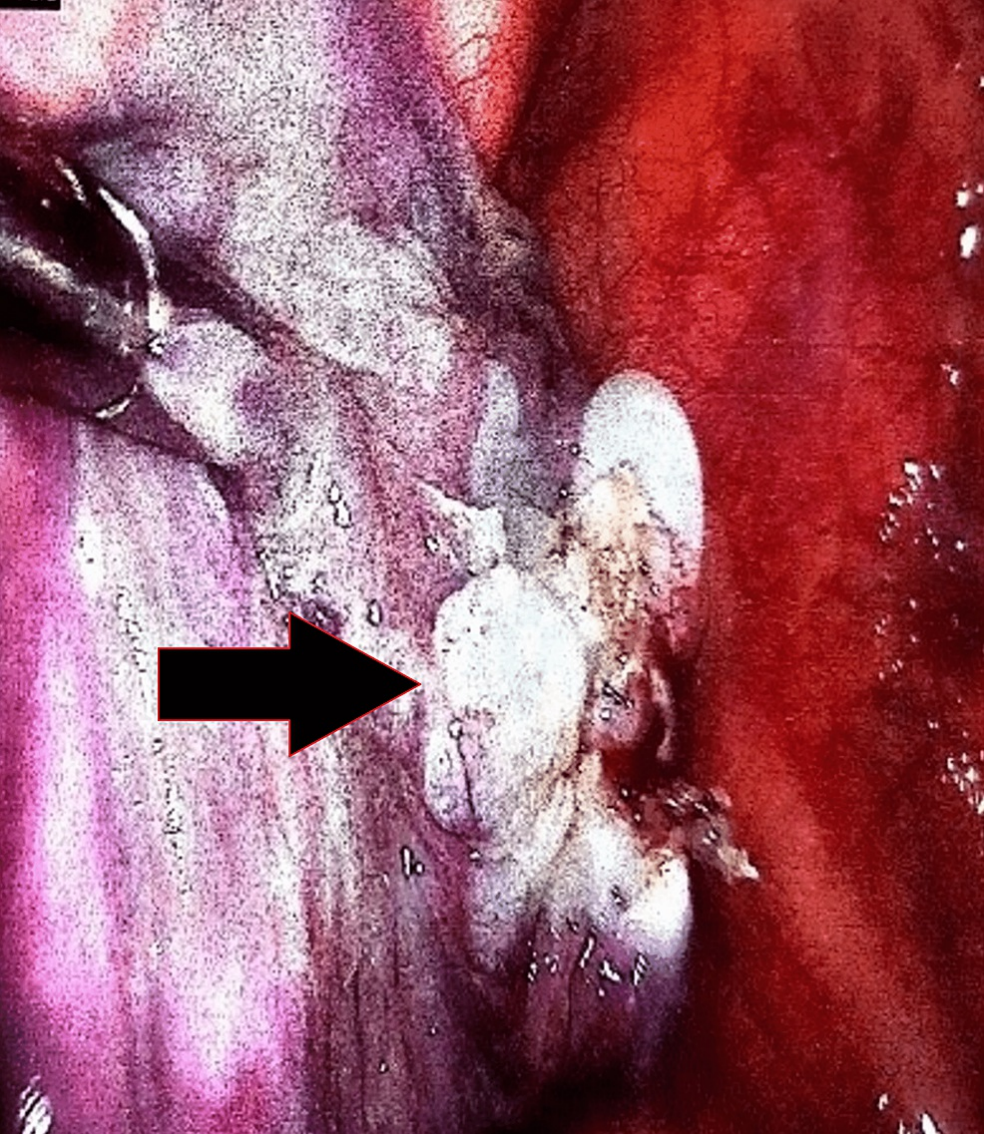
VATS showing multiple pneumatoceles tied with the help of loop cautery

The patient was extubated within 48 hours. He received antibiotics, ceftriaxone, and oxygen therapy. After 11 days of ICD insertion, the right lung had expanded entirely, and it was removed. On the left side, there was persistent pneumothorax. Hence, video-assisted thoracic surgery (VATS) was performed (Figure 5).

**Figure 6:**
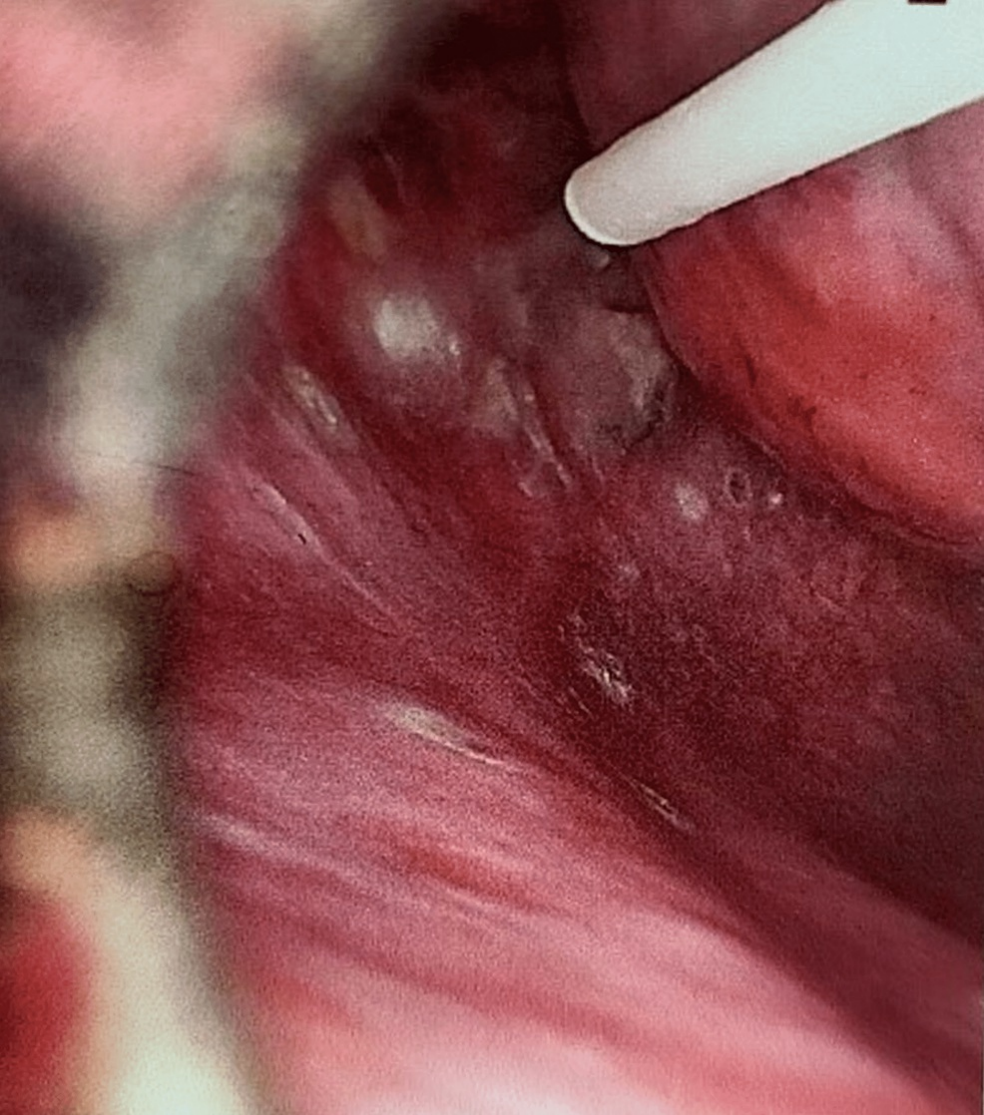
Pleural abrasion done with abrasion cautery

**Figure 7:**
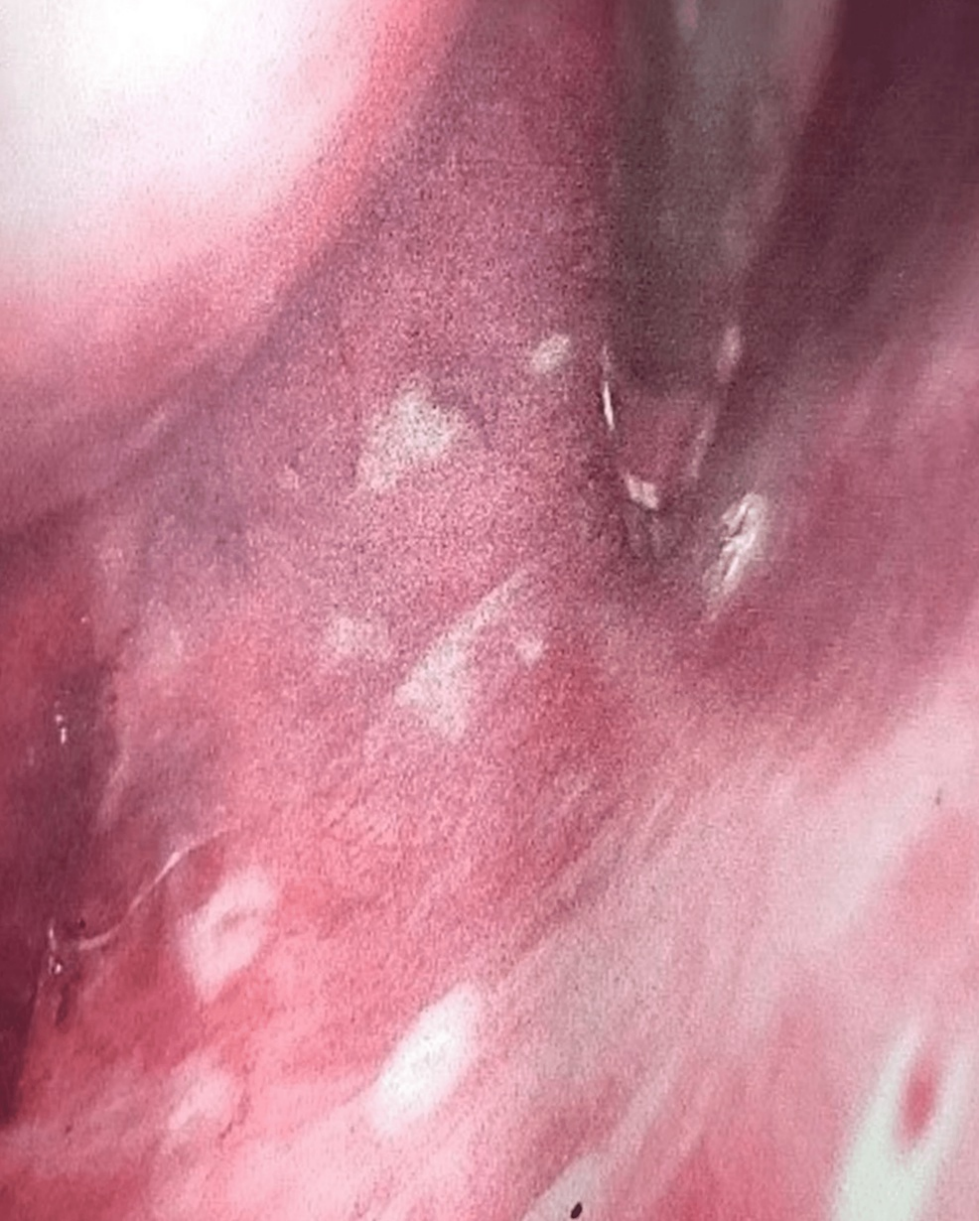
Pleurodesis achieved by talc poudrage

Under general anaesthesia, endotracheal intubation was performed. Then, during VATS, three large bullae on the left lung were resected. Mechanical abrasion of the parietal pleura was done using electrocautery (Figure 6). Pleurodesis was achieved by talc poudrage; 28 ICDs were inserted and fixed in situ (Figure 7).

The procedure was uneventful. Bronchoalveolar lavage was done during the procedure, which was positive for Mycobacterium tuberculosis using a line probe assay using the GenoType MTBDR plus kit, a molecular genetic assay for identification of resistance to rifampicin and/or isoniazid in the Mycobacterium tuberculosis complex, and was sensitive to both rifampicin and isoniazid. Alpha-1 antitrypsin level was 1.62 gm/l (normal:-0.9-2.0 gm/l) and was done to rule out emphysema from Alpha-1 antitrypsin deficiency. The patient was discharged on an anti-tubercular regimen, and after six months, the patient completed the AKT4 and is doing well.

## Discussion

Pneumothorax appears spontaneously or follows trauma to the chest [1].

Pneumothorax is classified as tension or non-tension. A tension pneumothorax is a medical emergency as an increase in intrathoracic pressure due to air accumulation in the pleural space causes circulatory and/or respiratory failure. Circulatory collapse is caused by mechanical mediastinal compression, and respiratory failure is caused by lung collapse and decreased air exchanges [2]. Our patient had both a respiratory and a circulatory collapse.

Spontaneous pneumothorax is also differentiated as primary or secondary. Primary spontaneous pneumothorax (PSP), defined as a pneumothorax without underlying lung disease, predominantly occurs in young, thin males. Ruptured pleural blebs, or bullae, usually cause it. PSP might be associated with congenital disorders like Marfan’s syndrome or behavioural risk factors like smoking. PSP usually occurs at rest. There are some precipitating factors, such as a change in atmospheric pressure or emotional instability [3].

The simultaneous bilateral spontaneous pneumothorax incidence has been reported as 1.4%-7.6%[1-3]. Primary spontaneous pneumothorax frequently affects young, tall, and thin males [1]. Our patient was also young, tall, lean, and thin. Wilder et al. stated in 1962 that 78% of the cases of simultaneous spontaneous secondary pneumothorax studied were due to tuberculosis (TB), suggesting 1.4% of TB patients have this complication [4]. The above-mentioned case can be counted as a part of both primary and secondary bilateral spontaneous pneumothorax, as the patient had multiple subpleural bullae and tuberculosis. Other causes of simultaneous bilateral spontaneous pneumothorax include pulmonary metastases, histiocytosis X, undefined interstitial pulmonary disease, sarcoidosis lymphangioleiomyomatosis, rheumatoid arthritis, scleroderma, ankylosing spondylitis, Marfan syndrome, Ehlers-Danlos syndrome, necrotizing pneumonia, and pneumocystis carinii pneumonia [5].

Patients usually complain of chest pain, followed by a sudden shortness of breath. The severity of the symptoms depends on the extent of the pneumothorax. In unilateral pneumothorax, pneumothorax can be confirmed immediately and precisely by clinical examination. Tactile vocal fremitus and breath sounds are diminished, and there is hyperresonance on percussion at the affected side. In bilateral pneumothorax, the severity of dyspnoea is increased to such an extent that the patient can collapse hemodynamically [2]. In such cases, a bilateral silent chest with hyperresonance can be observed.

Management of pneumothorax

Emergency treatment includes needle aspiration followed by percutaneous catheter drainage or intercostal drain insertion [1]. The surgical management of pneumothorax is indicated in bronchopleural fistula with air leakage even after ICD insertion, recurrent pneumothorax on the same side, simultaneous bilateral pneumothorax, pneumothorax developed after pneumonectomy, and pneumothorax with an occupational cause [2,4]. In our case, the patient developed a persistent bronchopleural fistula on the left side for 14 days. Hence the decision to implement VATS was taken. Video-Assisted Thoracic Surgery (VATS) is one of the most common forms of treatment modality used for the management of persistent pneumothorax [3]. Alternative procedures are possible (transaxillary mini-thoracotomy (TAMT), mechanical pleurodesis, chemical pleurodesis, etc.). Usually, bilateral pneumothorax requires definitive surgical therapy to reduce recurrence [3,6]. This can be achieved through VATS or open thoracotomy followed by mechanical pleurodesis to reduce the recurrence rate. The above-mentioned case presented a life-threatening situation of simultaneous bilateral spontaneous pneumothorax, which was treated with immediate large-size needle decompression at the emergency room, followed by intercostal drainage and VATS. Our report is the first case where bilateral spontaneous pneumothorax occurred due to both primary (sub-pleural bleb) and secondary causes (tuberculosis).

Role of the ventilator in pneumothorax

Our patient, who was in severe respiratory distress, was intubated early, within ten minutes of admission to the Medicine Intensive Care Unit (MICU). In the MICU, pneumothorax results from barotrauma due to mechanical ventilation, thoracocentesis, and central venous line insertion. Before putting the patient on a mechanical ventilator, we should rule out pneumothorax, as mechanical ventilation can exacerbate pneumothorax. Our patient on admission had bilaterally diminished breath sounds and a hyper-resonant note suggesting he had bilateral pneumothorax prior to mechanical ventilation [7].

Other complications are the re-expansion of pulmonary oedema that may occur in some PSP patients after undergoing chest tube insertion and drainage procedures. This rare but life-threatening complication arises from rapid pulmonary re-expansion after decompression. Young age, significant and persistent (usually >24 h) pneumothorax and rapid expansion of the lung are risk factors for this complication.

## Conclusions

This is a unique case of simultaneous spontaneous bilateral pneumothorax caused by tuberculosis and subpleural bullae. Initially, the patient was treated with a bilateral intercostal drainage tube, followed by video-assisted thoracoscopic surgery and pleurodesis. Pneumothorax should be kept as a first differential diagnosis in a life-threatening case of sudden onset dyspnoea. Once it is diagnosed early, treatment with a simple procedure like a needle thoracotomy can save the patient's life.
